# Towards sustainable performance of urban horticulture: ten challenging fields of action for modern integrated pest management in cities

**DOI:** 10.1007/s41348-020-00379-x

**Published:** 2020-09-22

**Authors:** Falko Feldmann, Ute Vogler

**Affiliations:** Institut für Pflanzenschutz in Gartenbau und Forst – Julius Kühn-Institut, Messeweg 11-12, 38104 Braunschweig, Germany

**Keywords:** Urban horticulture, Integrated pest management, IPM in cities

## Abstract

We identified ten current key challenges for plant protection in cities each of them belonging to a specific field of action of IPM in urban horticulture according to Directive 2009/128/EC. The challenges are: appropriate plant selection, microbiome engineering, nutrient recycling, smart, digital solutions, diversification of vegetation, avoidance of pesticide side effects on beneficials, biorational efficacy assessment, effective pest diagnosis, efficient outbreak control and holistic approaches. They are discussed on the background of the defined urban horticultural core sectors (a) public green infrastructure, including professional plant care, (b) professional field and greenhouse production systems and (c) non-professional private homegardens and allotments.

## Introduction

Urban horticulture is one of the most important socio-economic sectors for future city designs (Edmondson et al. 2020) combining economical, ecological and societal demands. The balance of these demands leads to sustainability. Production of plants used in cities, including food, is re-discovered in city planning currently. Being part of typical cities for centuries and forgotten over decades, more and more city designers have space in mind for horticultural plant production (Edmondson et al. 2020).

As city, we understand urban areas including the closer peri-urban space. While the Food and Agriculture Organization of the United Nations defines peri-urban agriculture as "agriculture practices around cities which compete for resources (land, water, energy, labour) that could also serve other purposes to satisfy the requirements of the urban population (FAO 1998), we here share the conceptual view of the Nottingham and Liverpool Universities (1998). The peri-urban interface is generally considered as a transitional zone between city and countryside, often described “not [as] a discrete area, but rather [as] a diffuse territory identified by combinations of features and phenomena, generated largely by activities within the urban zone proper”. For us, therefore, the plant production zone with direct contact to the city belonging to a pre-urban transitional zone is called peri-urban zone. One of the characteristics is the direct marketing of fresh produce on local markets in the city.

Urban horticulture provides cultivated plants for the city and secures their sustainable use, including plant care. The recycling and post-use fate of horticultural plants defines the borderline to urban bioeconomy based on higher plants. Urban horticulture covers a large spectrum of plant uses from food (fruits, vegetables, medicinal plants), to ornamentals, and structural elements in the green urban infrastructure. Natural elements of the spontaneously growing vegetation are part of urban horticulture because of their detrimental effects as weeds or their beneficial ecosystem services. Unlike urban agriculture, urban horticulture focusses on plant production and use only except in dual or tripartite production systems.

In contrast to arable farming, in urban horticulture a huge amount of cultivated plant species and cultivars of different growth form types are produced and utilized, for instance trees, bushes, forbs, herbs, annuals and even fungi. Plant production takes place in open fields, greenhouses, on roof tops, sky farms (Germer et al. 2011), at house walls, fallow gaps between buildings (Anderson and Minor 2017), and inside of houses, including private and public buildings (Nwosisi and Nandwani 2018). Public parks and public open spaces are even thought as places for food production (Casalegno et al. 2017), e.g. in “edible city concepts” (Bohn and Viljoen 2010). Plant cultivation might be carried out in a large scale and in small scale in homegardens or allotments. Tree care as part of urban horticulture is very important and mainly focused on public areas.

**Fig. 1:**
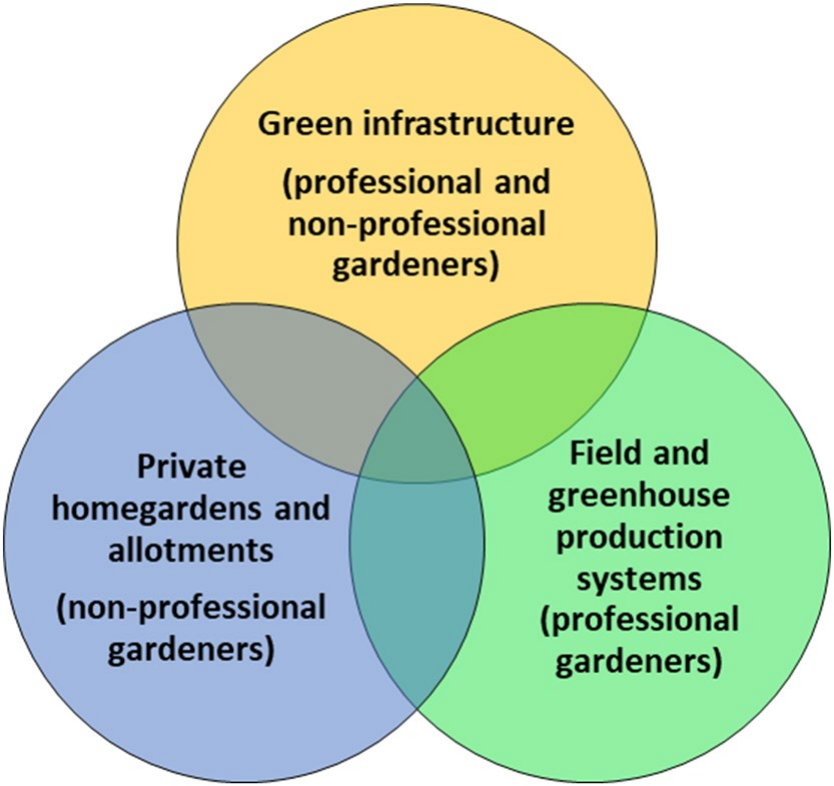
Core sectors of plant production and plant use in urban horticulture

Urban horticultural plants may be grown by professional and non-professional gardeners. Professional gardeners have a special education managing plant production including plant protection. Non-professional hobby gardeners might have traditional or no specific knowledge in plant production including plant protection. Public areas, private gardens and field and greenhouse production areas, including plant nurseries, form the green infrastructure and are the core sectors of urban horticulture (Fig. 1).

**Fig. 2:**
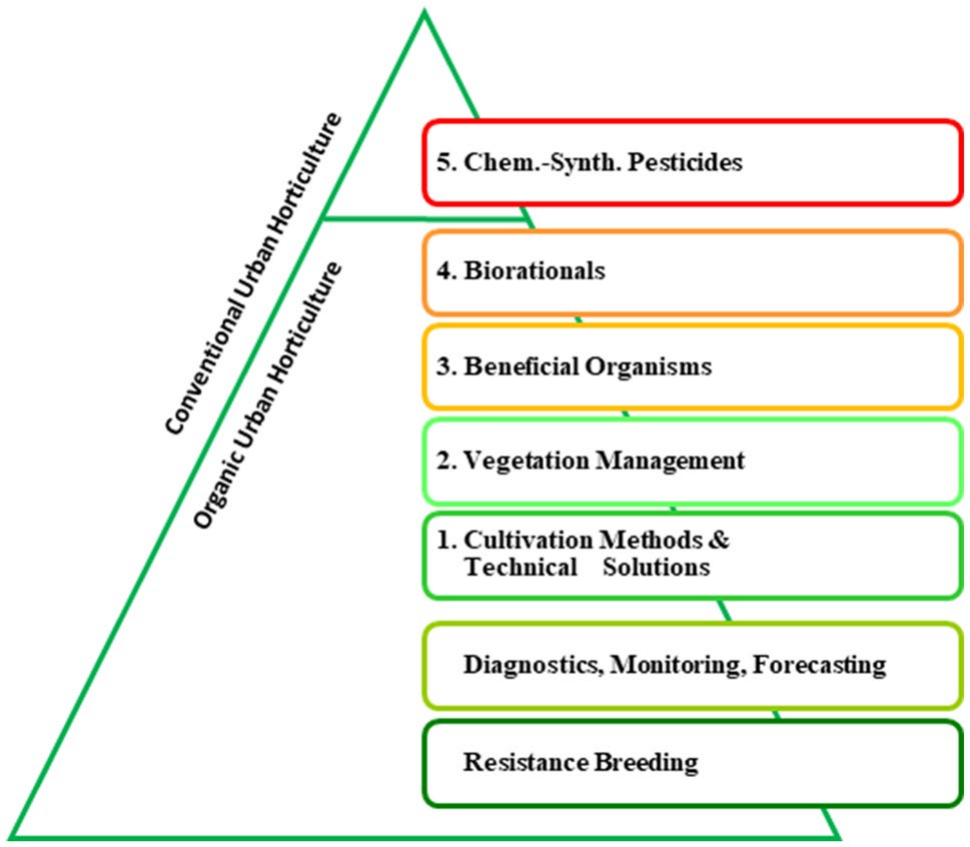
Integrated Pest Management according to Directive 2009/128/EC, Annex III (Preventive and protective steps 1 to 4 are possible in both, organic and conventional urban horticulture, step 5 only in conventional urban horticulture)

The broad spectrum of plant species requires a large variety of plant protection strategies, which have to be adapted to diversified scenarios of plant production and plant use. All plant protection strategies should follow the strategic concept of Integrated Pest Management (IPM, Fig. 2) according to Directive 2009/128/EC, Annex III (European Parliament and Council 2009). The IPM outlined in this directive defines the use of chemical-synthetic pesticides as final option after unsuccessful or inefficient integration of all other measures. Consequently, IPM is often confronted with societal demands for plant production with a partial to total abandonment of high risk pesticides and the production of residue-free products, which should at the same time protect natural resources significantly.

Research and development permanently provides new information about the biology of pests, appropriate monitoring and forecasting systems, prophylactic and direct plant protection measures, risk mitigation strategies and technological solutions. More and more the combination of different techniques in IPM takes place. Against this background, we identified ten current challenges for IPM from recent literature, each of them belonging to a specific field of action of IPM in urban horticulture according to Directive 2009/128/EC, Annex III (European Parliament and Council 2009). They will be discussed on the background of the defined urban horticultural core sectors.

### Challenge 1: Appropriate plant selection for urban horticulture

The plant varieties cultivated in professional horticulture mainly are determined by demands of retailers. Currently, quality criteria are characteristics important for climate adaptation, growing and cropping conditions, long distance transport, assumed consumer demands like size, taste characteristics, absence of diseases symptoms. Retail dominates the market for horticultural products and restricts or widens the spectrum of offers and the prices.

Direct marketing will change all these circumstances significantly. Urban horticulture will lead to a re-definition of quality characteristics of plants produced locally. Plants with short shelf life can enter the market because product distribution is direct, without retail and rapid. The interrelationship between product quality and production quality (Feldmann 2007) demonstrated by producers directly to the consumer will rise the understanding for certain IPM practices and will transparently discuss risks and chances of management options. This can dramatically change the spectrum of plants cultivated in or around cities. Already nowadays, there are examples, which show a new “sociotechnical landscape” (Hosseinifarhangi et al. 2019) based on glasshouse production in cities.

Plant selection for production in urban glasshouses will concentrate on plants with a short shelf life like herbs, salads, soft fruits and ornamentals. A huge number of such plants and cultivars are already available. It depends on the technical standard of the greenhouses how the IPM system has to be designed and which plants could be grown (Zeidler et al. 2019). Technical solutions of IPM will ease the adaptation of plants to greenhouse production and offer options to select new plants for the market.

For open field production, the use of cultivated plant varieties resistant to abiotic and biotic stresses is a pre-requisite for the reduction in chemical-synthetical pesticides and other plant protection measures. Vegetable crops are of major importance for peri-urban and urban food production. In vegetable crops, resistant cultivars are almost the only possibility of directly controlling phytopathogenic viruses, bacteria and nematodes. While there are plant protection products available for several pathogenic insects, mites and fungi, for a number of pathogens no active substances are available neither for professional nor for non-professional use. In these cases, breeding is the only option to prevent against these hazards.

Thus, resistance breeding is an important pillar in crop protection in horticulture, e.g. in vegetables. For each host–pathogen system, an individual breeding program is necessary followed by the combining resistance genes afterwards. Resistance breeding is, therefore, very time-consuming and expensive. Breeding companies rely on basic research to adopt new findings into their resistance breeding strategy to develop new resistant cultivars. Joint approach of breeding research and resistance breeding in the breeding company is the prerequisite for efficiency. In addition to the classical resistance breeding, the cross-breeding of resistance genes or resistance alleles in current breeding material are of interest as well as resistance breeding by genetic engineering methods with genetic engineering of resistance genes from other species and genera, as well as the modification, supplementation and optimization of resistance associated genes, e.g. by *Agrobacterium* transfer, Direct GT, RNA interference, genome editing, cisgenics, CRISPR/Cas and others. It is a challenge to focus breeding research on resistance to highly potent pathogens, to species previously underrepresented in resistance breeding, and to new, potentially invasive pathogens and quarantine pathogens (Nothnagel et al. 2019). Evaluation of quantitative traits can be supported by modern sensing technologies for precision phenotyping (Tripodi et al. 2018).

Plants in the green infrastructure, including trees, are not only faced with biotic stresses caused by herbivores and pathogens, but also with abiotic stresses like heat and drought. For instance, tree characteristics are relevant for their suitability to cool heat stands in cities (Rahman et al. 2020). Here, vulnerability assessments to environmental factors like climate change have to be carried out in situ and in experimental gardens (Ordóñez and Duinker 2014). Such analyses have to consider already the nursery production systems. They have a significant influence to the survival of trees in the urban environment (Allen et al. 2017).

The challenge in this field of action mainly is to sample all the experiences made worldwide or on national, and regional level. Information systems have to be established to ease the exchange of observations including climatic, edaphic and utilization parameters. National databases may contain information in different languages, but should minimally provide an English abstract. Peer reviewed journals should publish reviews and meta-analyses based on national languages in English to make the data internationally available. Existing databases may be interconnected and opened (e.g. www.deutsche-genbank-obst.de), and existing signets should be included (e.g. www.adr-rose.de/).

### Challenge 2: Microbiome engineering

Since several years, on each level of plant production, whether professional or non-professional, special attention is paid to natural microbiome engineering in cultivation systems. In urban areas, most plants are produced in technologically processed cultivation materials or in disturbed soils. Under such conditions, normally a lack of beneficial microorganisms exist in the substrates and can create suboptimal growth and increase in diseases (Feldmann 1998). The current challenge is to acquire the positive effects of microorganisms associated with plants for production and utilization systems (Pandit et al. 2020). The urban growing conditions, which are mainly substrate-based, offer an ideal laboratory to test microbiomes under both professional and non-professional practical conditions.

The totality of microorganisms in a certain environment, e.g. a plant, a plant surface, in the rhizosphere, etc., is called a microbiome. Microbiomes have a considerable potential for crop protection. They can naturally lead to systemically acquired resistance and induced systemic resistance, altering the metabolism of the plant at many levels, reducing stress and detoxifying the cells (Brader et al. 2017). The targeted use of these microbiomes is influenced by management, i.e. use of pesticides, fertilizers, etc., the plant genotype and the developmental stage of the plants, as well as other climatic and biotic environmental conditions (Saikkonen et al. 2020). The conducive conditions of organic farming have already shown great advantages to this regard (Muller et al. 2017). While seed coating with microorganisms is already a good method for delivering beneficial microbes to crops (Rocha et al. 2019), a practical challenge is the complexity of microbiomes (Sessitsch et al. 2019). The understanding on the fate of inoculants and on interactions between plants, and the environment, under field conditions is still limited (Mitter et al. 2019), and a lot of microorganisms remain still uncharacterized (Freimoser et al. 2016).

Other, symbiotic cultivation measures have a further effective contribution to crop protection of horticultural crops and can be modulated (Pascale et al. 2019). Mutualistic symbioses of root-colonizing mycorrhizal endophytes are used to improve plant nutrition, plant health, plant development and plant quality in horticulture since decades (Feldmann 1998). The provision of nutrients not available for plants, changes of the phytohormone balance, antagonistic activities, influence on resistance and tolerance of the plant and even positive effect on the physico-chemical properties of the soil are detectable in numerous examples and can often be achieved through directed inoculum production (Feldmann et al. 2009).

In the future, natural microorganism communities, microbiomes including symbiotic mycorrhizal fungi can hopefully be stabilized resulting in new products used in horticultural production (Bitterlich et al. 2018).

### Challenge 3: Nutrient recycling

Non-professionals within cities have a nutrient regeneration challenge: they want to produce from their growing systems and, therefore, have to revolve nutrients from other sources (Jezik and Bauer 2001). To gain nutrients from external resources is a delicate, difficult and under-estimated problem. Solutions for urban horticulture are seen in symbiosis with decentralized waste management strategies (Weidner and Yang 2020). These strategies may provide the peri-urban production with new sources for nutrients, too, if they avoid competition with electricity generation. Cities, which are processing and selling compost, offer an effective way of nutrient recycling already today.

Generally, appropriate fertilization is a widely overseen pre-requisite for the growth of healthy plants. Effects of mulch and nitrogen fertilizer on the soil environment of crop plants is recognized by conventional horticultural production being interested in reducing costs by utilizing ecosystem services. The use of mulch in horticultural field production can influence the microclimate, in detail the evaporation from the soil, the relative humidity and the temperature and microbial biomass in the soil. Furthermore, the use of mulch maintains soil organic carbon balance, optimises nutrient cycling, promotes soil enzyme activity, enhances soil aggregate stability and suppresses weed infestation (Wang et al. 2019). This favours crop plant development.

Recognizing this, practices are integrating mineral fertilization and biofertilization, including animal manure and horn shavings (Sangeeth and Suseela Bhai 2016) with the tendency to reduce mineral fertilization. Mineral nitrogen fertilization is not applied in organic horticultural production systems, if emphasis is laid on proper nitrogen regeneration through rotation systems. The substitution of the regeneration phase by mineral fertilization facilitated the establishment of plant pests and plant diseases followed by insufficient pathogen control and pesticide use. (Junge et al. 2017). Due to the natural activity of symbiotic nitrogen fixers, the temporal change of extraction and regeneration phases can dispense mineral fertilizers and at the same time promote the development of a diverse soil microbiome (Geisseler et al. 2017). The combination of mulching and crop rotation will not only reduce pathogen pressure. Together with tillage systems (Alliaume et al. 2017), the whole integrative approach opens opportunities for peri-urban production.

### Challenge 4: Smart solutions for IPM in cities

Digitalisation of integrated pest management in cities is a challenge, which can provide several advantages for the user: professional and non-professional gardeners receive quick and precise information and decision support what should be done where and when. Expert knowledge and consultancy on IPM measures, diagnostic help and documentations can be made available via digitalisation. Vice versa civil scientific approaches can increase data quantity and quality on diseases or pest outbreaks in urban areas and can result in new ways of warning systems.

Digitalisation of IPM in cities could be put forward easily today (OECD 2020). It can fall back on a lot of options and smart solutions already developed. Furthermore, especially in cities the internet of things (IoT) including sensor networks, which integrate single measurements to smart systems via artificial intelligence (AI), can be expanded easily because of existing infrastructural pre-requisites. Wireless sensor networks together with spatial decision support systems, and satellite communication can be integrated to support IPM (Petric et al. 2018). Even monitoring of pathogens can be taken over by smart agro-robotic solutions (Grieve et al. 2019). Such IoTs can be installed in open urban spaces, for instance, by automating weather forecasting, soil moisture measurements, water harvesting and irrigation.

Gardeners can be involved via IoT by urban gardening mobile apps. The capabilities offered by smart sensing and data science, new opportunities to carry out large-scale studies are created involving social science and human factors (Ferrara et al. 2018). Such apps are available for a wide variety of users, allowing them to collect data and often directly evaluate them. In an effort to become more resilient and contribute to saving water and other resources, people become more interested in growing their own food, but do not have sufficient gardening experience and education on conserving water including water harvesting and water recycling. IoT-based mobile apps are developed for this purpose as decision support systems (Penzenstadler et al. 2018). The “Garden App” shows gardeners the role of their garden in the green network of the city (Schneider et al. 2020). With further apps like the “Bee App”, the user receives advice how to turn his garden with trees, bushes or annuals more bee-friendly (Federal Ministry for Food and Agriculture Germany 2020). In addition, beneficial resource tools or guidebooks for detecting and remedying plant damage are offered. In the phytosanitary sector, interactive apps enable automatic photo-identification of pests or the option to send in the photograph and have it evaluated by experts. For the future, artificial intelligence is planned to assume the identification of pests and pest symptoms autonomously.

Overall, the potential for using apps is enormous. The automatic image recognition is getting better, smartphones are everywhere, some portals already have over 1 million users. But: to maintain apps useful is a permanent task, both in terms of design, features, content and updating. The effort is often carried out by companies to promote their products. A longer-term financing and establishment of non-profit Apps from public side is therefore necessary. Above all, it requires the appreciation for app development within science. Journals would have to rise a focus on this and establish recognition.

A first important step would be to make existing, not protected data available to cities, local authorities and universities free of charge and to eliminate the lack of data availability. This would lead, for instance, to the opportunity to map cities based on multi-criteria approaches ensuring urban ecosystem demands (Li et al. 2020).

For urban vegetable and ornamental production indoor, in greenhouses and outdoor, but also for tree growth observations in the public green infrastructure there is a spectrum of options for sensor-assisted detection, description and forecasting of diseases and pests in urban horticulture. Based on the knowledge of the biology of the pathogens, the clear description of symptoms and the quantification of the symptoms including the damage threshold of crops allows a large number of applications (Keszthelyi et al. 2020). Among the measuring methods and sensors already in use today are RGB and false colour cameras including computer image processing, hyper and multispectral cameras (Thomas et al. 2018), UV, VIS and NIR spectroscopy, chlorophyll fluorescence spectroscopy (Dadras Javan et al. 2019) or image analysis, thermal imaging cameras (thermography), wetness sensors (Ehlert et al. 2019) electro-chemical sensors, electronic nose VOCs (Sun et al. 2019) and acoustics in storage pests (Banga et al. 2018). In the future, pest search and automated sensor phenotyping of plant populations may also be advantageous (Thomas et al. 2018). However, to detect and to recognize harmful organisms and their damage to horticultural crops in an early stage of development is still a challenge. While optical processes and traps are the most advanced, other existing detection methods would have to be further developed and new methods explored.

The "Phyto-control" system in greenhouses (Miranda et al. 2017) opened a further new direction of research. Sensors detect signals of the crop plants and transform the signals for automated precise control of the microclimate in greenhouses: Phytometric, elektrophysiological information ensures healthy and vital plant populations (Tran et al. 2019). However, knowledge of the needs of the plant population and the exact determination of the climatic conditions in the greenhouse make it already possible to avoid climatic risk areas through control measures. This avoids climatic ranges that increase phytosanitary risk. Additionally, the protection and promotion of beneficial organisms is possible.

Light-based control of herbivorous insects in horticulture utilizes the visual behaviour of herbivorous insects (e.g. white fly in greenhouses). The migration of insects can be induced by light, but also repellent and attractant effects were observable. LED-enhanced panels were very successful to catch detrimental insects, light barriers could reduce infestation (Stukenberg and Poehling 2019). Combinations of green and UV LEDs can be used to modify the attractiveness of plants by initiating flight activity. Reliable automatic image processing has now to be developed and the embedding of the methods in robotics and automation has to be carried out. UV B and UV A radiation are effective to alter the secondary metabolism of the plant (Rechner et al. 2017). Indirect effects on the insects may be conceivable by light-induced increase in resistance. Unfortunately, negative effects can occur when useful insects are attracted at the same time like pests. Outdoor and in glasshouse on roof tops in urban areas “light smog” have to be avoided because of such negative side effects.

### Challenge 5: Diversification of urban vegetation

All over the world, large city planning recognizes the high value of ecosystem services resulting from turning grey infrastructure to green. Vegetation management in cities is one of the most challenging aspects designing this green infrastructure. Ecosystem services by plants are thought to support health of citizens, e.g. by reducing heat in city centres (Li et al. 2020). On the other hand, the question arises how control loops between plants belonging to the green infrastructure can be used in IPM and whether biodiversity of these plant communities supports health of plants as well.

Up to date, plant diversity and composition in private urban plant communities are still driven by horticultural availability of plants and homeowner preferences (Cavender-Bares et al. 2020) and not by ecological considerations. Moreover, expectations and imaginations of visitors of public parks, including leisure demands, are often in conflict with ecologically driven design of modern parks considering ecosystem services (Talal and Santelmann 2020).

Besides this social complication of vegetation management in cities, information about horticulturally influenced natural control loops in urban plantings are scarce in spite of the fact that biocontrol of pathogens in IPM bases on natural regulatory cycles (Balder 2002). In cities, ecological services in the sense of biological regulation between arthropod predators and prey exist (Gardiner et al. 2014), even belowground interrelationships may be used in urban gardens (Yadav et al. 2012). In a large scale, environmental modifications of park vegetation led to a diversification of functional biodiversity (Czortek and Pielech 2020). But in small scale there are only rare examples of sufficiently working control mechanisms due to vegetation management in cities. Flower strips, often used as biocontrol elements in horticulture (Snyder 2019), are currently studied with respect to biodiversity of pollinators (Hicks et al. 2016) but could be used for multiple IPM uses (Balzan et al. 2016). In agricultural environments, semi-natural vegetation supported sustainable establishment of useful spiders in viticulture (Kolb et al. 2020) and the securing of the availability of suitable and sufficient floral biodiversity was found to be a pre-requisite for natural enemies of apple pathogens (Herz et al. 2019).

For planting trees in urban green infrastructure including streets and parks, artificial substrates often are preferred instead of soil, roots are directed and water resources are connected with tree stands. Therefore, trees often stand “alone” and only later some ground covers are additionally planted. To overcome this substrate-dependent mono-plant system, new strategies are suggested, e.g. the so-called urban forest management, which tries to mix vegetation of different growth form types in cities (Nitoslawski et al. 2019). Here, an open challenge remains to adapt co-planted plants to substrates favourable for trees, to optimize growing conditions and microclimate for better co-plant and tree growth and to promote resilience towards changing climatic conditions especially in urban/ peri-urban ecosystems.

With regard to this field of action in urban green infrastructure, a further new strategy may open new possibilities: cross-kingdom communication via applied chemical ecology with special regard to “infochemicals”.

Infochemicals are active substances used as semiochemicals and innovative strategies for sustainable and environmentally sound pest control in agriculture. Interspecifically, effective allelochemicals such as allomones, kairomones and synomones and intraspecifically pheromones are used to influence the behaviour of insect pests and thus to protect crops. Applied technologies deliver selectively effective repellents, develop dispensers, nanotechnology and microencapsulated repellents.

But infochemicals exist liberated by naturally growing plants as well. More and more it becomes clear that there is localized defence induction in plants caused by herbivory of insects. This results in a mosaic of leaf traits promoting variation in plant traits, predation and communities of canopy arthropods (Volf et al. 2020). Volatile organic compounds seem to be reliable indicators of insect herbivory (Griese et al. 2017) and may function as infochemicals about attractiveness of plants or working as repellents. Phytochemical diversity drives plant–insect community diversity (Richards et al. 2015), and plant/pests have to be seen as communication network (Vogler et al. 2010).

In basic research, researchers turn to study chemically mediated interspecific signalling pathways and the impact of climate change on the chemical communication of insects (Gross et al. 2019) and plant–insect combinations. Infochemicals as well as plants acting via infochemicals are promising areas of this research. Insects may be lured to targets for ecosystem services, for instance, for selective pest control using their attractants. Innovative monitoring tools, microencapsulated attractive infochemicals for attracting opponents together with microorganisms or nematodes killing them (Jaffuel et al. 2019) open up numerous opportunities for the development of combined strategies such as push and pull, attract and kill and push–pull kill strategies. This is a huge challenge and potential for vegetation composition in urban environments.

### Challenge 6: Avoidance of side effects on beneficials

As already mentioned above, beneficial meso-organisms (mainly arthropods and nematodes) provide important ecosystem services for useful plants growing in production systems or in the urban green infrastructure. Biological crop protection with beneficial arthropods is a standard procedure in vegetable cultivation in greenhouses, e.g. growing cucurbitaceae, solanaceae and herbs (Richter 2009). The same is true for many ornamental plants, e.g. for garden and balcony plants, poinsettias, cyclamen, roses, gerberas and others. But the use of beneficial organisms could be considered in much more plants in both, protected and open field cultivation, including non-professional homegarden production systems.

Disadvantages of the use of beneficial organisms are still the more complicated application, low control thresholds, a high demand of consultation, training needs, but also, that the plants might be not completely clean and free of diseases symptoms and pests. Nevertheless, the use of beneficial organisms will expand, as fewer and fewer crop protection products are available. Even if the use of beneficial organisms requires system adaptation during operation, the benefits are increasingly recognized: There are less impacts on the environment, no chemical pesticide residues in food, no user risk, no waiting period, sustainable, long lasting efficacy, no resistance and pollination by bumblebees will be possible. Procedures should be as standardized as possible and improve practicability through bio-integrated approaches. There is a particular need for research in biorationals and plant activator application and their compatibility with beneficial organism use, as well as studies of field applications.

A complementary demand would be the declaration of IPM fitness of pesticides on their labels to show the compatibility with beneficials (Böckmann et al. 2019).

### Challenge 7: Biorational efficacy assessment

A further escalation step of IPM is the direct control of pests by biorationals, most of which are allowed for use in plant protection of organic agriculture according to Commission Regulation (EC) 889/2008 (EU Commission 2008).

In the course of a two-tiered approval process (European Parliament and of the Council 2009), chemical-synthetical agents can be classified as low-risk plant protection products. Together with microbiological pesticides, botanicals (plant extracts), plant activators (substances that protect plants by activating their defence mechanisms) and basic substances (often agents that are also available on the market as food ingredients, these different groups, which bear a lower risk for health and environment, are together named “biorationals” (Feldmann and Carstensen 2018; Matyjaszczyk 2018). Added to these biorationals are biostimulants belonging to the fertilizer legislation and containing a number of beneficial microorganisms (European Parliament and Council 2019), e.g. mycorrhizal fungi. The introduction of such alternatives for direct control is made even more difficult by their unpredictable efficacy: the efficacy of these alternative products is only tested in the case of microorganisms, plant extracts and low-risk chemical-synthetical agent. They may be approved with sufficient effectiveness, but also with less efficacy. In the case of basic substances, only a "useful plant protection effect" is demonstrated without any quantification of effectiveness. In any case, the measured efficacy is transparently labelled.

In order to be able to make meaningful use of low-risk resources, the following could solve this challenge: (a) authorities should be able to make the scope and outcome of their efficacy assessment transparent, e.g. on the label of the products; (b) applicants should be able to promote the integration of their products into integrated pest management concepts; (c) horticultural demonstration enterprises should be promoted and collaborate with research organizations to develop novel, integrated plant protection strategies; (d) plant protection services should include low risk resources in their audits and policy developments; (e) all should learn to benefit from the experience of organic plant protection and be more closely linked to a common integrated approach to crop protection; and (f) relevant stakeholders should work to strengthen the importance of evaluating the effectiveness of "lower risk" products.

### Challenge 8: Effective pest diagnosis

Rapid, correct identification of pathogens, especially new pathogens, is an important challenge in all urban horticultural core sectors. Here, the large quantity of pathogens and cultivated plants make it difficult to recognize especially new pathogens at a stage before a major outbreak. Sufficient experts, e.g. taxonomists, are of major importance. Furtherly, modern methods of diagnosis are necessary for their support.

High‐throughput identification techniques can offer the basis for early warning systems. In a very detailed review (Tedersoo et al. 2019), all relevant high throughput methods are summarised: Quantification methods like qPCR, droplet digital PCR, spiking combined with high-throughput sequencing (HTS) are standard approaches, which still have a variety of applications in the future and hopefully can be used for the routine detection of mixed samples of different pathogens. Microarray methods enabled targeting specific pre‐selected taxa of viruses, bacterial and fungal pathogens and pests at species level, but are more and more replaced by HTS methods. HTS methods are mainly used for the identification of species. They include nanopore technologies, which are promising because of low cost. Further high throughput technologies evolving are rising from the field of metagenomics and meta-transcriptomics and are used for pathogen studies already. TEDERSOO et al. (2019) predict “that rapid monitoring methods such as nanopore sequencing, microarrays and nanotechnological biosensors will become particularly useful for early disease diagnostics and smart application of countermeasures such as biocides and biocontrol agents”. All these methods will ease monitoring in horticulture. But bioinformatics to analyse the data sets and databases for data storage and reporting have to be co-developed at the same time.

Rapid diagnosis is the pre-requisite for advisory services providing substantial information for professional and non-professional gardeners. Especially in cities, there is the need to develop information networks, which already exist in arable farming systems, for instance demonstration gardens (Dachbrodt-Saaydeh 2018). Advisory services should work independent and knowledge based. Because of the huge amount of private gardeners, new information systems could be combined with mobile plant health clinics as already working in countries outside of Europe (Tambo et al. 2020).

### Challenge 9: Efficient outbreak control

Within the IPM concept, chemical-synthetical pesticides are the final option to be chosen, when all prevention measures, including beneficials and biorationals, did not succeed. Chemical-synthetical pesticides are intensively studied and reviewed during the official approval process (European Parliament and of the Council 2009). Restrictions allow the secure application for eradication of severe pathogens. In cities, the application of such chemical-synthetical pesticides with acceptable risk is allowed in professional plant production systems while in public areas and private homegardens only low-risk products are allowed. As already described above, the advantage of chemical-synthetical pesticides is their relatively good predictable efficacy. Consequently, professional plant producers use them for outbreak control above certain threshold levels to avoid complete or partial loss of the produce. One of the major challenges to guarantee the future availability of such pesticides is the responsible use as final option after all the other steps of escalation of IPM. Already now, for several plant/pathogen or pest combinations in professional horticulture no efficient plant protection product is available.

A further challenge is to make effective pesticides rapidly available for new emerging pest outbreaks. As an example, a dramatic outbreak of the quarantine bacterium *Xylella fastidiosa* decimating olive production in the European Union (EU) was discovered in 2013 in Apulia, Southern Italy (Saponari et al. 2019). Even after years, no efficient control of this disease or its vector is possible. Still the solution might be the occurrence of resistant olive trees, while the bacterium is spreading. Further hope rises from a computational biology approach using tools for identifying specific ligand binding residues for novel pesticide design (Neshich et al. 2015). This example demonstrates that rapid development of specifically acting chemical-synthetical substances and rapid screenings of existing active substances against the bacteria or the vectors are urgently needed to counteract severe pest outbreaks.

Outbreak control in cities is much more complicated than in arable farming systems. The cities are an accumulation of so-called bystanders, citizens who could come in contact with the pesticides used in urban horticulture. Therefore, pesticides are specifically approved for public areas and special technologies are used for direct control. For instance, the Oak Processionary Moth (*Thaumetopoea processionea*) or the Asian Longhorn Beetle (*Anoplophora glabripennis*) need an appropriate management (Monteiro et al. 2019). In cities even more than in agriculture, precise application of minimum doses without drift to the environment have to be further developed, e.g. drone use. (Meyer 2016).

The examples show that rapid diagnosis together with effective pesticides and efficient application technology has to be developed for the urban environment, which is appropriate for the control of outbreaks of emerging pathogens and, at the same time, is not harmful for the citizens.

### Challenge 10: Holistic approaches

#### IPM

IPM as described here and as laid down in Directive EC 2009/128 is thought as a combination of escalating plant protection measures starting from plant selection upwards to the use of the necessary amount of chemical-synthetic pesticides. In urban environments, it becomes obvious that IPM should be a holistic approach: here, all components have to be established at the same time and all of them are intimately interconnected and explicable by reference to the whole, the healthy plant. In this approach, the use of chemical-synthetic and biological pesticides are restricted to certain uses, e.g. to control the outbreak of pests. Research on the challenging fields of action as listed above would allow to improve the connections between the components of IPM in cities.

IPM is the most important factor of plant production (compare Matyjaszczyk 2019). Modernization of IPM in cities is characterized by fostering the “harmless” components and the reduction in direct control measures. Optimization of planting site factors via vegetation management, including creation of interconnectivity, will lead to diversified, complex combinations of cultivated plants with natural spontaneously growing plant elements, together called guilds and will result finally in an efficient green urban infrastructure. Consequently, growing food in cities will avoid monocultures. Trees, bushes, herbs, all growth form types will be mixed to guilds, in which all components support each other. This brings biodiversity back to cities if the IPM is appropriate (Nicholls et al. 2020).

#### Food production and distribution

Food production in cities is assumed to have several important aspects, including shortening of transport chains and enhancing awareness of citizens for food value. A considerable part of vegetables might be produced here (Martellozzo et al. 2014), and trees may play an important role in stabilizing the urban food system (Park et al. 2019) if they are not in competition with urban forests (Richardson and Moskal 2016). City food production is, therefore, part of the strategies to feed the world (Muller et al. 2017), especially in times of crisis like the Covid-19-pandemy (FAO 2020).

The digitisation of horticulture as shown above does not only offer new opportunities for professional gardeners. Much more applications are available for city gardeners than for farmers in agriculture. In cities, traditional horticultural knowledge and high-tech options will be combined and lead to smart cities. In such smart cities, for instance, water management in gardens or parks can be regulated easily by adequate sensor techniques. Nutrient cycles can be closed by such techniques as well.

Such developments ease plant production for non-professionals, too. Another holistic challenge rises in strengthening of non-professional citizens’ responsibility for food production and distribution.

If more parts of the green infrastructure can be used for food production, all citizens have to be involved to a certain extent. Digitalisation will allow the coordination of complex approaches like IPM.

Information networks have to be constructed and food chains become food networks as well. Information should be bi-directional: on the one hand, information about new production techniques is distributed; on the other hand, experiences about cultivars used, quantities harvested or times of harvest and allocation chains will be redistributed. This will create a completely different awareness of food value and city environment value in urban gardeners and related consumers. Even more, if the process is accompanied by scientists, a very fruitful development of citizen science approaches will be possible.

#### Permaculture

Citizen-based food production does currently not follow certain concepts but is very variable. Non-professional plant protection does often not follow the idea of IPM (Nicholls et al. 2020). Since several years, the idea of permaculture is spreading and reaches city gardeners as well (Ferguson and Lovell 2014). The concept is basically driven by mixed cultivation of different cultivated plant species and cultivars, often in guilds, using hugel culture or other nutrient cycling planting systems. Following the idea of plant protection, all the components presented as challenges for IPM in this contribution are realized in a functioning permaculture. Permaculture might even act as link between professionals and non-professional food production because there are the first examples of professional permaculture companies in the peri-urban area. Moreover, there are arguments both for the importance to understand permaculture as a social safety-net and as experimental testing ground for cutting edge biomimetic technologies. The small scale ecological system-design of permaculture might finally serve as a model of a future agricultural paradigm (Stojanovic 2019).

#### Outlook

Planning towards productive performance and ecological contributions of urban horticulture, the open questions remain: how can all core sectors in urban horticulture be integrated? How much food can we then produce in cities and which quality will the goods have? How can we measure production in such complex situations like permaculture scientifically? How should we develop the food distribution network in cities including non-professional gardeners?

Answering these questions will open further challenging fields of action in urban horticulture. But IPM will be the basis for its sustainability.

## Data Availability

All data used are cited under the reference chapter.
